# Darker Skin Color Measured by Von Luschan Chromatic Scale and Increased Sunlight Exposure Time Are Independently Associated with Decreased Odds of Vitamin D Deficiency in Thai Ambulatory Patients

**DOI:** 10.1155/2021/8899931

**Published:** 2021-02-28

**Authors:** Nipith Charoenngam, Sutin Sriussadaporn

**Affiliations:** ^1^Vitamin D,Skin and Bone Research Laboratory, Section of Endocrinology,Nutrition,and Diabetes, Department of Medicine, Boston University Medical Center, Boston, MA, USA; ^2^Division of Endocrinology and Metabolism, Department of Medicine, Faculty of Medicine Siriraj Hospital, Mahidol University, Bangkok, Thailand

## Abstract

**Background:**

Little is known about the association among skin color, sunlight exposure. and vitamin D status in Southeast Asian population.

**Objective:**

To investigate the association between skin color measured by von Luschan chromatic scale (VLCS) and vitamin D status in Thai medical ambulatory patients.

**Methods:**

Medical ambulatory patients were enrolled. The eligibility criteria were as follows: aged >18 years, stable medical conditions, and no conditions directly affecting vitamin D status. Serum 25-hydroxyvitamin D [25(OH)D] levels were assessed. Skin color at the outer forearm was assessed using VLCS which grades skin color from the lightest score of 1 to the darkest score of 36. Patients were systematically interviewed to estimate daily sunlight exposure time.

**Results:**

A total of 334 patients were enrolled. Data were expressed as mean ± SD. The mean serum 25(OH)D was 25.21 ± 10.06 ng/mL. There were 17 (5.1%), 217 (65.0%), and 100 (29.9%) patients who had light brown (VLCS score 18–20), medium brown (VLCS score 21–24), and dark brown (VLCS score 25–27) skin colors, respectively. The mean serum 25(OH)D level was higher in patients with dark brown skin than in patients with medium brown and light brown skin (28.31 ± 10.34 *vs.* 24.28 ± 9.57 and 19.43 ± 9.92 ng/mL, respectively, both *p* < 0.05). Multivariate analysis showed that darker skin color and increased sunlight exposure time were independently associated with decreased odds of vitamin D deficiency (dark brown vs. light brown: odds ratio, 0.263, 95% CI: 0.081–0.851, *p*=0.026; medium brown vs. light brown: odds ratio, 0.369, 95% CI: 0.987–1.003, *p*=0.067; sunlight exposure time odds ratio per 1 minute/day increase 0.955, 95% CI: 0.991–1.000, *p*=0.037), after adjusting for possible confounders.

**Conclusions:**

We found that darker skin color at sunlight exposure area and increased sunlight exposure time were independently associated with decreased odds of vitamin D deficiency in Thai medical ambulatory patients.

## 1. Introduction

Vitamin D is a steroid hormone responsible for regulating calcium and phosphorus metabolism and maintaining healthy mineralized skeleton [1, 2]. Humans get vitamin D from sunlight exposure, diets, and supplements. There are two forms of vitamin D, including vitamin D_2_ (ergocalciferol) and vitamin D_3_ (cholecalciferol). Vitamin D_2_ is synthesized from ergosterol and found in yeast and mushrooms. Vitamin D_3_ is endogenously synthesized in the skin and found naturally in animal products [1, 2]. Once vitamin D enters the circulation, it is metabolized by the enzyme 25-hydroxylase in the liver to 25-hydroxyvitamin D [25(OH)D], which is then converted by the enzyme 25-hydroxyvitamin D-1*α*-hydroxylase in the kidneys into the active form, 1,25-dihydroxyvitamin D [1,25(OH)_2_D]. 1,25(OH)_2_D binds to vitamin D receptor in various tissues to exert its physiologic functions [2, 3]. Endogenous vitamin D_3_ synthesis requires exposure of the skin to ultraviolet B (UVB) radiation. Factors influencing cutaneous vitamin D_3_ synthesis include: the dosage of UVB radiation in viable wavelength of 290–315 nm exposed to the epidermis, the amount of 7-dehydrocholesterol, the vitamin D_3_ substrate, in the skin, and skin pigmentation [1, 2, 4].

It has been shown that individuals with constitutionally black or darkly pigmented skin such as in African population require a larger amount of UVB exposure to synthesize an equivalent amount of vitamin D compared with those with lightly pigmented skin, leading to a higher risk of vitamin D deficiency [5–8]. However, more recent studies have suggested that skin color and sunscreen use do not significantly affect vitamin D synthesis and that only minimal amount of UVB exposure is likely sufficient to maintain sufficient vitamin D status [9, 10]. Additionally, darkening of the skin might reflect skin tanning as a result of repetitive sunlight exposure especially in non-black populations with lighter skin colors [11]. Repetitive sunlight exposure, however, has been shown to upregulate the expression of melanogenic proteins and cause the expansion of melanin containing melanocytic processes towards the skin surface, which might consequently block the penetration of UVB radiation into the epidermis [11]. Whether and how skin tanning affects the efficacy of cutaneous vitamin D synthesis or is associated with vitamin D status in constitutional non-black individuals is still to be clarified.

A number of previous observational studies aiming to identify the association between skin color and vitamin D status have been conducted in different ethnic populations with different constitutional skin colors and geographic residency areas bathed with varying amounts of sunlight such as European [12–18], North American [19], Latin American [20], Australian [6, 21–23], and Middle Eastern populations [24]. However, the results of these studies are markedly inconsistent, and only few of them did assess sunlight exposure of their participants and include this factor into their analysis [14, 15]. To the best of our knowledge, there is no study on the association among skin color, sunlight exposure time, and vitamin D status in South East Asian population in which most people have constitutional non-darkly pigmented skin color [25].

**Figure 1:**
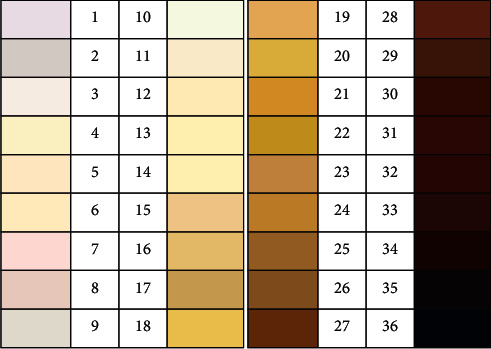
Von Luschan chromatic scale (reference 28).

Von Luschan chromatic scale (VLCS) (Figure 1) is a practical tool for measurement of skin color of which the score has been shown to highly correlate with that measured by the gold standard method, reflectance spectrophotometry [26]. The current study was therefore conducted with the aim to investigate the relationship among vitamin D status, sunlight exposure time, and skin color measured by VLCS [27] and to examine whether skin color can be used as an index for determining the vitamin D status in Thai population in which most people have constitutional non-darkly pigmented skin color.

## 2. Material and Methods

### 2.1. Patient Recruitment

This cross-sectional study randomly recruited adult medical ambulatory patients who regularly attended the outpatient clinic of the Division of Endocrinology and Metabolism, Department of Medicine, Faculty of Medicine Siriraj Hospital Mahidol University, Bangkok, Thailand (1.5 meters above sea level, coordinate 13°45′ N 100°29′ E) for ongoing treatment. The study protocol was approved by the Siriraj Institutional Review Board (SIRB) (COA no. Si 163/2016). This study complied with the principles set forth in the Declaration of Helsinki (1964) and all of its subsequent amendments. Written informed consent was obtained from all participating patients.

Each eligible participant was reviewed for medical history and current medication and was comprehensively interviewed for health status, daily activities, any possibility of receiving direct or indirect vitamin D supplement, and estimated daily sunlight exposure time. Participants who were eligible for this study must have all of the following inclusion criteria: (1) adult medical ambulatory patients older than 18 years of age; (2) stable medical conditions; and (3) able to perform general daily indoor and outdoor activities. Patients who had one or more of the following conditions were excluded: (1) diseases or conditions known to affect vitamin D metabolism, including liver diseases defined by serum glutamic oxaloacetic transaminase and serum glutamate-pyruvate transaminase of >3 times of the upper normal limit, severe kidney diseases defined by estimated glomerular filtration rate (eGFR) of <30 mL/min/1.73 m^2^ calculated by the Chronic Kidney disease Epidemiology Collaboration equation [28], overt hyperparathyroidism or hypoparathyroidism, untreated hyperthyroidism, inadequate or excessive thyroxine replacement, inflammatory bowel diseases, intestinal malabsorption, chronic diarrhea, current anticonvulsant therapy, corticosteroid therapy, receiving all forms of vitamin D supplement, and inability to perform normal daily activities; (2) diseases or conditions that affect skin color, including skin pigmentation disorders, generalized eczema, adrenal insufficiency, ACTH-producing tumors and Cushing disease, and hemochromatosis; (3) unable to recall or report estimated daily sunlight exposure time.

Initially, the medical record of patients who had appointments for the biweekly outpatient clinic during the study period (December 2016–May 2017) was preliminarily reviewed to determine the eligibility. Simple randomization was then performed to identify ten patients per clinic day to be the candidate participants of the study. Further screening interview was performed to verify that all patients fulfill the eligibility criteria.

### 2.2. Serum 25-Hydroxyvitamin D Measurement

Serum 25(OH)D levels were measured by electrochemiluminescence immunoassay (ECLIA) using an Elecsys 2010 automated immunoassay analyzer (Roche Diagnostics, Risch-Rotkreuz, Switzerland) that measures both 25-hydroxyergocalciferol (25(OH)D_2_) and 25-hydroxycholecalciferol (25(OH)D_3_). Results were reported in nanograms per milliliter (ng/mL). All serum 25(OH)D measurements were performed in a laboratory accredited by the International Organization for Standardization (ISO 15189), and were monitored using the Randox International Quality Assessment Scheme (RIQAS). Serum 25(OH)D levels of ≥30, 20–<30 and <20 ng/mL were defined as vitamin D sufficiency, insufficiency, and deficiency, respectively [29].

### 2.3. Estimation of Average Routine Daily Sunlight Exposure Time

Each patient was systematically interviewed to estimate average routine daily sunlight exposure time by one investigator (N. C.) using a questionnaire of which the contents had been validated before applying to this study (Table 1). Patients were asked to estimate average sunlight exposure time within separated 2-hour periods of each weekdays (Monday to Friday) and weekend (Saturday and Sunday) under their routine activities and clothes, including 6.00–8.00 a.m., 8.00–10.00 a.m., 10.00 a.m.–12.00 p.m., 12.00–2.00 p.m., 2.00–4.00 p.m., and 4.00–6.00 p.m. The average routine daily sunlight exposure time was calculated by summation of all the estimated sunlight exposure time for each 2-hour period.

### 2.4. Measurement of Skin Color

VLCS score, which semiquantitatively grades skin color with a wide range of color score from the lightest of 1 to the darkest of 36 (Figure 1) [26, 27], was used for assessment of skin color by one investigator (N. C.) who was well trained and standardized by an experienced dermatologist for how to use the VLCS chart. The skin color was assessed at the outer forearm that is the common sunlight-exposed skin area [30]. This area represents the combined effects of constitutional skin color and skin tanning. Skin color at the inner upper arm, which represents solely constitutional skin color, was also assessed. In order to determine the appropriate cut-off values to categorize the study participants' skin colors, a pilot study in 100 Thai ambulatory patients was conducted at our institute. Based on our finding that VLCS score ranged from 18 to 27 in the pilot patients, we classified the VLCS score into three groups with similar ranges of <21, 21–24, and ≥25, which were defined as light brown, medium brown, and dark brown skin colors, respectively.

### 2.5. Sample Size Calculation

There has been no previous study that evaluated the association between skin color measured by the VLCS and vitamin D status. In our study, sample size was calculated based on our pilot data in 100 patients and the primary objective of the study, which is to evaluate whether there was a significant difference in rate of vitamin D deficiency between groups with different skin colors (light brown *vs*. dark brown skin colors). According to our pilot study in 100 patients, we found that the rates of vitamin D deficiency in those with light brown and dark brown skin colors were approximately 75% and 26%, respectively. Therefore, at least 13 patients per group were required to achieve the statistical power of 80% with type 1 error of 0.05 in order to demonstrate the difference between groups. Since there were 4% of patients with light brown skin in the pilot data, at least a total of 325 patients were required to achieve at least 13 patients with light brown skin color.

### 2.6. Statistical Analysis

Results are expressed as number of subjects with percent (%) or mean ± standard deviation (SD) or standard error of the mean (SEM) as appropriate. Serum 25(OH)D levels, serum PTH levels, and sunlight exposure time were analyzed by using repeated-measures analysis of variance (ANOVA) to identify difference among groups of different skin colors. A Bonferroni post hoc *t* test was used to identify pairwise difference between groups of different skin colors. Adjusted odd ratios were estimated to determine the associations between skin colors and rate of vitamin D deficiency by using multivariable logistic regression analysis with inclusion of covariate terms including: age; sex; body mass index; presence of underlying diseases: diabetes mellitus, hypertension, dyslipidemia, and coronary artery disease; and eGFR. We did not include sunlight exposure time in the multivariate analysis because we expected that both vitamin D status and skin color would be highly dependent on this variable, and our aim is to investigate the possibility to use skin color as a marker of sunlight exposure to determine the odds of vitamin D deficiency. All statistical analyses were performed using a Statistical Package for the Social Sciences (SPSS) version 25.

## 3. Results

Initially, 500 adult ambulatory patients were randomly identified from the medical record. A total of 166 patients were excluded as they reported to take vitamin D supplementation. Finally, 334 adult medical ambulatory patients fulfilled the eligibility criteria and were included in this study. The patient characteristics are shown in Table 2. The mean age was 64.54 ± 10.45 years, and 214 cases (64.1%) were female. The mean body mass index (BMI) was 26.53 ± 4.04 kg/m^2^. The patients' major underlying diseases included diabetes mellitus (71.0%), hypertension (70.1%), dyslipidemia (82.6%), and coronary artery disease (7.8%). The mean eGFR was 72.79 ± 21.06 mL/min/1.73 m^2^. Serum 25(OH)D levels was 25.21 ± 10.06 ng/mL, and serum PTH was 49.05 ± 22.62 pg/mL. There were 25.5%, 41.9%, and 32.6% of the patients who had vitamin D sufficiency [25(OH)D ≥ 30 ng/mL], insufficiency [25(OH)D 20–<30 ng/mL], and deficiency [25(OH)D < 20 ng/mL], respectively. There were 17 (5.1%), 217 (65.0%), and 100 (29.9%) patients who had light brown (VLCS score of 18–20), medium brown (VLCS score of 21–24), and dark brown (VLCS score of 25–27) skin colors at outer forearm, respectively. There were 141 (42.2%), 183 (54.8%), and 10 (3.0%) patients who had light brown (VLCS score of 18–20), medium brown (VLCS score of 21–24), and dark brown (VLCS score of 25–27) skin colors at inner upper arm, respectively. Estimated daily sunlight exposure time of the patients was 63.54 ± 89.89 minutes per day (0–630 minutes). A total of 41 cases (12.3%), 55 cases (16.5%), and 238 cases (71.3%) used sunscreen every day or almost every day (6-7 days/week), frequently (3–5 days/week), and occasionally (<3 days/week), respectively (Table 2).

**Figure 2:**
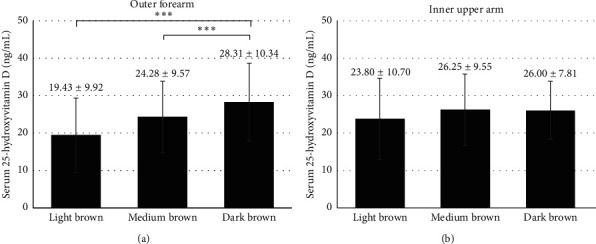
Serum 25-hydroxyvitamin D levels (ng/mL) in patients with light brown, medium brown, and dark brown skin colors at (a) outer forearm and (b) inner upper arm. Note: data were expressed as mean ± SD. “ ^*∗∗∗*^” denotes *p* < 0.005.

**Figure 3:**
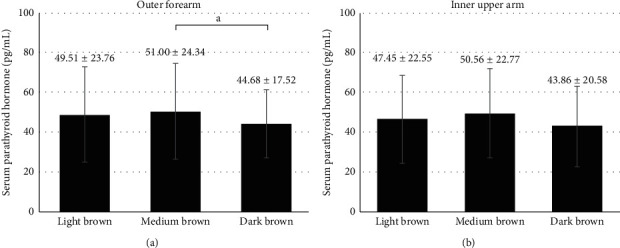
Serum parathyroid hormone levels (pg/mL) in patients with light brown, medium brown, and dark brown skin colors at (a) outer forearm and (b) inner upper arm. Note: data were expressed as mean ± SD. “a” denotes *p*=0.064.

**Figure 4:**
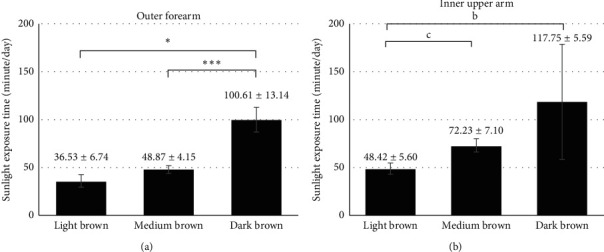
Estimated sunlight exposure time (minutes/day) in patients with light brown, medium brown, and dark brown skin colors at outer forearm and inner upper arm. Note: data were expressed as mean ± SD. “^∗^” denotes *p* < 0.05; “ ^*∗∗∗*^” denotes *p* < 0.005; “b” denotes *p*=0.053; “c” denotes *p*=0.052.

Comparisons among patients with dark brown, medium brown, and light brown skin colors at outer forearm are demonstrated in Figures 2–4. Serum 25(OH)D levels were higher in patients with dark brown skin color than in patients with medium brown and light brown skin colors (28.31 ± 10.34 *vs.* 24.28 ± 9.57 and 19.43 ± 9.92 ng/mL, respectively, both *p* < 0.05, Figure 2(a)). Patients with dark brown skin color at outer forearm tended to have lower serum PTH levels than patients with medium brown skin color (44.68 ± 17.52 *vs.* 51.00 ± 24.34 pg/mL, *p*=0.064, Figure 3(a)). Patients with dark brown skin color at outer forearm reported higher estimated daily sunlight exposure time than patients with medium brown and light brown skin colors (Mean ± SEM: 100.61 ± 13.14 *vs.* 48.87 ± 4.15 and 36.53 ± 6.74, respectively, both *p* < 0.05, Figure 4(a)). There was no statistically significant difference in serum 25(OH)D and PTH levels among groups with different skin colors measured at inner upper arm (Figures 2(b) and 3(b)), although patients with light brown skin colors tended to have higher estimated daily sunlight exposure time than the other two groups (*p*=0.05, Figure 4(b)).

Adjusted association of skin color at outer forearm and sunlight exposure time with odds of vitamin D deficiency is demonstrated in Table 3. Dark brown skin color was associated with decreased odds of vitamin D deficiency compared with light brown skin color (adjusted OR of 0.263, 95% CI: 0.081–0.851, *p*=0.026) after adjusting for age; sex; BMI; the presence of underlying diseases including diabetes mellitus, hypertension, dyslipidemia, and coronary artery disease; and estimated glomerular filtration rate. There was a trend towards statistically significant increased odds of vitamin D deficiency in patients with medium brown skin color compared with patients with light brown skin color (adjusted OR of 0.369, 95% CI: 0.987–1.003, *p*=0.067). A significant association between increased amount of sunlight exposure time and decreased odds of vitamin D deficiency was also observed (adjusted OR per 1 minute/day increase of 0.955, 95% CI: 0.991–1.000, *p*=0.037, Table 3).

## 4. Discussion

To the best of our knowledge, this is the first observational study aiming to determine the association among skin color at sun-exposed area, routine daily sunlight exposure time, and vitamin D status in Thai population, which is a representative for South East Asian population in which most people have constitutional non-darkly pigmented skin color. We have enrolled 334 medical ambulatory patients who had no vitamin D supplementation and conditions known to affect vitamin D status or skin pigmentation. The reason we conducted this study in the outpatients with chronic medical conditions was that they are expected to be more susceptible to vitamin D deficiency and its related consequences than the general population, and therefore evaluation and treatment of vitamin D deficiency would be of particular benefit in this population [31–34].

VLCS was used for semiquantitative measurement of skin color in this study as it is practical and the results have been shown to highly correlate with reflectance spectrophotometry which is the gold standard method used in the assessment of skin pigmentation [26]. As the skin area of outer forearm is the common sunlight-exposed area [30], it was used for assessment of the association among skin color in response to sunlight exposure, routine daily sunlight exposure time, and vitamin D status in this study.

We found that individuals with darker skin color at the outer forearm, which represents a sunlight-exposed area [30], had higher serum 25(OH)D levels than those with lighter skin color. In addition, the lower mean serum PTH level observed in individuals with dark brown skin color comparing to those with lighter skin color suggested the significant impact of the higher serum 25(OH)D levels or vitamin D status on parathyroid gland function. A similar dose-dependent association between darker skin color at sunlight exposure area and higher sunlight exposure time was also observed.

The observed dose-dependent association between darker skin color at sunlight exposure area and higher routine daily sunlight exposure time suggests that the association between darker skin color and increased serum 25(OH)D levels is likely mediated by the amount of individual routine daily sunlight exposure. Equally important is that the multivariable analysis revealed that darker skin color at the sunlight-exposed skin area and increased estimated routine daily sunlight exposure time were independently associated with decreased odds of vitamin D deficiency, after adjusting for age, sex, BMI, the presence of underlying diseases, and eGFR (Table 3). Although the exact mechanism of this observation is still unclear, it is probable that skin tanning at sunlight-exposed area might also represent the combination of intensity and repetition of sunlight exposure independent of the average routine daily sunlight exposure time.

It has been accepted that melanin pigment in the skin is a natural sunscreen that blocks the penetration of UVB radiation into the epidermis, leading to a decrease in cutaneous synthesis of vitamin D. Therefore, individuals with darkly pigmented skin require a larger amount of sunlight exposure to synthesize an equivalent amount of vitamin D compared with those with lightly pigmented skin, thereby having an increased risk of vitamin D deficiency [5, 35]. Nevertheless, recent studies have indicated that skin color and sunscreen use do not significantly affect endogenous synthesis of vitamin D and that only minimal amount of UVB exposure is likely adequate for an individual to be vitamin D-sufficient [9, 10]. Our findings, however, indicate that darkening of the skin at sun-exposed area in Southeast Asian individuals reflect skin tanning as a result of repetitive sunlight exposure, rather than representing the blocking effect of increased melanin pigment on UVB penetration required for vitamin D synthesis as previously observed in ethnic darkly pigmented individuals [5].

A number of previous studies have been conducted with the aim to determine the association between skin color and vitamin D status in various ethnic populations with different skin colors and geographic residency areas that are bathed with different amount of sunlight including European [12–18], North American [19], Latin American [20], Australian [6, 21–23], and Arabic [24] populations. However, only two previous studies included both skin color and sunlight exposure in the same analysis to determine the relationship with vitamin D status [14, 15]. Fitzpatrick skin phototype is the most frequently used tool of measurement of skin color among these studies [12–17, 20, 23], followed by spectrophotometer [6, 23] and reflectance colorimeter [19, 21]. There is a large discrepancy in the association between vitamin D status and skin color observed in those studies, probably due to difference in the background of the study populations as well as the methods of skin color assessment across the studies. Remarkably, most of the studies that reported the association between darker skin color and sufficient vitamin D status and/or higher serum 25(OH)D levels were conducted in Caucasian population [12–15], whereas the majority of the studies that reported the opposite included participants with various ethnicities [17–19].

Our observations may have clinical implications as they suggest that skin color at outer forearm along with self-reported sunlight exposure time obtained from systematic interview (Table 1) can be used for stratifying the risk of vitamin D deficiency in Southeast Asian population or may be used in other population with constitutional non-darkly pigmented skin color [6, 21, 22]. Several methods have been utilized for assessment of skin color. Fitzpatrick skin phototype, the commonly used method in most studies, is a subjective scale that classifies skin color into 6 types (I–VI) based on the constitutional skin colors before sunlight or UV exposure and the degree of reaction to sunlight or UV exposure [36]. Other less frequently used methods include reflectance spectrophotometry and reflectance colorimetry which quantitatively measure skin color as the individual typology angle (ITA) [37, 38] and the von Luschan chromatic scale (VLCS) score which semiquantitatively grades skin color with a wide range of color score from the lightest of 1 to the darkest of 36 [26]. VLCS serves different purposes in the assessment of skin color from Fitzpatrick skin phototype. The Fitzpatrick skin phototype is a self-reported tool that categorizes the skin into 6 types (I–VI) based on the constitutional colors before and tanning reaction after exposure to sunlight [25, 36]. It is therefore useful for determining sunburn risk and risk for skin cancer and nevertheless is highly dependent on race and genetic predisposition [25]. In contrast, VLCS can semiquantitatively measure the actual skin color at any interested skin areas, and the obtained skin color score can be used to classify the skin color with a wide range from the lightest score of 1 to darkest score of 36 [26]. VLCS offers several advantages over Fitzpatrick phototype in assessing skin color to determine its association with vitamin D status in our situation. First, the actual skin color at sun-exposed area represents the combined effects of constitutional skin color and skin tanning, both of which are associated with endogenous vitamin D production. Second, within a certain ethnic group such as Southeast Asian population, VLCS can classify individuals who have the same Fitzpatrick skin phototype into more diverse categories. Finally, it can be easily used by any health-care providers and does not rely on patient self-reports [26]. Therefore, assessment of skin tanning using VLCS, in addition to an interview for routine daily sunlight exposure time, might be an effective tool for stratifying the odds for vitamin D deficiency and may be useful for guiding the management in settings where serum 25(OH)D level testing is not generally available. Further studies are required in order to determine the effectiveness of VLCS and systematic interview for routine daily sunlight exposure time in assessing risk for vitamin D deficiency before it can be recommended in clinical practice.

This study carries some conceivable limitations and caution is needed for the interpretation and generalization of the results. First, all of the patients in this study were ambulatory medical patients with medical co-morbidities including diabetes mellitus, hypertension, dyslipidemia, and coronary artery disease that might directly affect vitamin D status. Therefore, our study population might not definitely represent the general Southeast Asian population. However, we included ambulatory patients with well-controlled medical conditions who were unlikely to have limited outdoor activity. In addition, the VLCS of our study participants was in the same range as that observed in our pilot study and a previous report [39]. Thus, the observed association between skin color and vitamin D status would be comparable to that of the general population. Second, ascertainment of sunlight exposure time was based on patient self-reports, and accurate information on the intensity of UVB that the participants are exposed to is not available. This could jeopardize the reliability of the data. However, all patients were systematically interviewed by one investigator, thereby facilitating patients' recall and eliminating interobserver variation. Finally, since the cut-off values of VLCS have never been reported before, we specified the range of skin color as “light brown,” “medium brown,” and “dark brown” by using our predetermined criteria based on the data from our pilot study. Further studies are required to confirm the reliability of these cutoff values in stratifying the risk of vitamin D deficiency.

## 5. Conclusion

Using the VLCS to assess skin color and systematic interview to assess daily sunlight exposure time, we found that darker skin at the sun-exposed area is associated with increased sunlight exposure time, higher serum 25(OH)D levels, lower serum PTH levels, and lower odds of vitamin D deficiency in Thai medical ambulatory patients. In addition, darker skin color and increased sunlight exposure time were independently associated with decreased odds of vitamin D deficiency. We propose the use of skin tanning assessment by VLCS along with a systematic interview for sunlight exposure time as an index for measurement of repetitive sunlight exposure and for stratifying the risk of vitamin D deficiency which may be useful for guiding the management in Southeast Asian populations, especially in the settings where serum 25(OH)D level testing is not generally available.

## Figures and Tables

**Table 1:** Questionnaire for ascertainment of daily sunlight exposure time.

Day of week	6 am–8 am	8 am–10 am	10 am–12 pm	12 pm–2 pm	2 pm–4 pm	4 pm–6 pm
Question: In your usual clothes and on average, how many minutes are you exposed to sunlight during each specified period?						

Sunday						
Monday						
Tuesday						
Wednesday						
Thursday						
Friday						
Saturday						

**Table 2:** Patient characteristics.

Patient characteristics (*N* = 334)		
Age (years).	64.54 ± 10.45	
Female sex	214 (64.1%)	
Sunscreen use		
Rarely (<3 days/week)	238 (71.3%)	
Occasionally (3–5 days/week)	55 (16.5%)	
Every day/almost every day (6-7 days/week)	41 (12.3%)	
Body mass index (kg/m^2^)	26.53 ± 4.04	
Underlying diseases		
Diabetes mellitus	237 (71.0%)	
Hypertension	234 (70.1%)	
Dyslipidemia	276 (82.6%)	
Coronary artery diseases	26 (7.8%)	
eGFR (CKD-EPI, mL/min/1.73 m^2^)	72.79 ± 21.06	
Serum 25(OH)D (ng/mL)	25.21 ± 10.06	
Vitamin D status		
25(OH)D ≥30 ng/mL	85 (25.5%)	
25(OH)D 20–<30 ng/mL	140 (41.9%)	
25(OH)D <20 ng/mL	109 (32.6%)	
Serum PTH (pg/mL)	49.05 ± 22.62	
Von Luschan chromatic scale score	Outer forearm	Inner upper arm
*Light brown (VLCS score <21)*	17 (5.1%)	141 (42.2%)
18	0 (0%)	3 (0.9%)
19	1 (0.3%)	35 (10.5%)
20	16 (4.8%)	103 (30.8%)
*Medium brown (VLCS score 21*–*<25)*	217 (65.0%)	183 (54.8%)
21	44 (13.2%)	55 (16.5%)
22	59 (17.7%)	35 (10.5%)
23	41 (12.3%)	59 (17.7%)
24	73 (21.9%)	34 (10.2%)
*Dark brown (VLCS score 25–<27)*	100 (29.9%)	10 (3.0%)
25	60 (18.0%)	8 (2.4%)
26	26 (7.8%)	1 (0.3%)
27	14 (4.2%)	1 (0.3%)
Daily sunlight exposure time (min/day)	63.54 ± 89.89	

Data are expressed as mean ± SD or number of patients (percentage) as appropriate. Abbreviations: eGFR: estimated glomerular filtration rate; 25(OH)D: 25-hydroxyvitamin D; PTH: parathyroid hormone; VLCS: von Luschan chromatic scale score.

**Table 3:** Adjusted association of skin colors at the outer forearm and sunlight exposure time with odds of vitamin D deficiency (25-hydroxyvitamin D <20 ng/mL).

	Adjusted OR	95% confidence interval	*p*-value

Age (per 1 year increase)	0.990	0.960–1.020	0.500
Sex			
Male	Ref	Ref	Ref
Female	0.455	0.254–0.813	0.008
Body mass index (per 1 kg/m^2^ increase)	1.094	1.025–1.167	0.007
Skin color at outer forearm			
Light brown (VLCS score <21)	Ref	Ref	Ref
Medium brown (VLCS score 21–<25)	0.369	0.987–1.003	0.067
Dark brown (VLCS score 25–<27)	0.263	0.081–0.851	0.026
Sunlight exposure time (per 1 minute/day increase)	0.995	0.991–1.000	0.037
Type 2 diabetes mellitus	0.848	0.453–1.589	0.608
Hypertension	1.788	0.986–3.241	0.056
Dyslipidemia	1.399	0.703–2.783	0.339
Coronary artery disease	0.526	0.201–1.376	0.190
Estimated glomerular filtration rate (per 1 Ml/min/1.73 m^2^ increase)	1.003	0.987–1.018	0.737

Ref: reference; VLCS: von Luschan chromatic scale.

## Data Availability

The data used to support this study are available upon reasonable request to the corresponding author.

## References

[1] Wacker M., Holick M. F. (2013). Sunlight and vitamin D. *Dermato-Endocrinology*.

[2] Holick M. F. (2007). Vitamin D deficiency. *New England Journal of Medicine*.

[3] Charoenngam N., Shirvani A., Holick M. F. (2019). Vitamin D for skeletal and non-skeletal health: what we should know. *Journal of Clinical Orthopaedics and Trauma*.

[4] Chen T. C., Chimeh F., Lu Z. (2007). Factors that influence the cutaneous synthesis and dietary sources of vitamin D. *Archives of Biochemistry and Biophysics*.

[5] Clemens T. L., Henderson S. L., Adams J. S., Holick M. F. (1982). Increased skin pigment reduces the capacity of skin to synthesise vitamin D3. *The Lancet*.

[6] Dix C. F., Bauer J. D., Martin I. (2017). Association of sun exposure, skin colour and body mass index with vitamin D status in individuals who are morbidly obese. *Nutrients*.

[7] Harris S. S. (2006). Vitamin D and African Americans. *The Journal of Nutrition*.

[8] O’Connor M. Y., Thoreson C. K., Ramsey N. L. M., Ricks M., Sumner A. E. (2013). The uncertain significance of low vitamin D levels in African descent populations: a review of the bone and cardiometabolic literature. *Progress in Cardiovascular Diseases*.

[9] Young A. R., Narbutt J., Harrison G. I. (2019). Optimal sunscreen use, during a sun holiday with a very high ultraviolet index, allows vitamin D synthesis without sunburn. *British Journal of Dermatology*.

[10] Hawk J. L. M. (2020). Safe, mild ultraviolet-B exposure: an essential human requirement for vitamin D and other vital bodily parameter adequacy: a review. *Photodermatology, Photoimmunology & Photomedicine*.

[11] Tadokoro T., Yamaguchi Y., Batzer J. (2005). Mechanisms of skin tanning in different racial/ethnic groups in response to ultraviolet radiation. *Journal of Investigative Dermatology*.

[12] Malvy D. J.-M., Guinot C., Preziosi P. (2000). Relationship between vitamin D status and skin phototype in general adult population. *Photochemistry and Photobiology*.

[13] Glass D., Lens M., Swaminathan R., Spector T. D., Bataille V. (2009). Pigmentation and vitamin D metabolism in Caucasians: low vitamin D serum levels in fair skin types in the UK. *PLoS One*.

[14] Andersen L. B., Abrahamsen B., Dalgård C. (2013). Parity and tanned white skin as novel predictors of vitamin D status in early pregnancy: a population-based cohort study. *Clinical Endocrinology*.

[15] Emmerson A. J. B., Dockery K. E., Mughal M. Z., Roberts S. A., Tower C. L., Berry J. L. (2018). Vitamin D status of white pregnant women and infants at birth and 4 months in North West England: a cohort study. *Maternal & Child Nutrition*.

[16] Brembeck P., Winkvist A., Olausson H. (2013). Determinants of vitamin D status in pregnant fair-skinned women in Sweden. *British Journal of Nutrition*.

[17] Richard A., Rohrmann S., Quack Lotscher K. C. (2017). Prevalence of vitamin D deficiency and its associations with skin color in pregnant women in the first trimester in a sample from Switzerland. *Nutrients*.

[18] Santos A., Amaral T. F., Guerra R. S. (2017). Vitamin D status and associated factors among Portuguese older adults: results from the Nutrition UP 65 cross-sectional study. *BMJ Open*.

[19] Au L. E., Harris S. S., Dwyer J. T., Jacques P. F., Sacheck J. M. (2014). Association of serum 25-hydroxyvitamin D with race/ethnicity and constitutive skin color in urban schoolchildren. *Journal of Pediatric Endocrinology & Metabolism: JPEM.*.

[20] Cabral M. A., Borges C. N., Maia J. M., Aires C. A., Bandeira F. (2013). Prevalence of vitamin D deficiency during the summer and its relationship with sun exposure and skin phototype in elderly men living in the tropics. *Clinical Interventions in Aging*.

[21] Rockell J. E. P., Skeaff C. M., Williams S. M., Green T. J. (2008). Association between quantitative measures of skin color and plasma 25-hydroxyvitamin D. *Osteoporosis International*.

[22] Cargill J., Lucas R. M., Gies P. (2013). Validation of brief questionnaire measures of sun exposure and skin pigmentation against detailed and objective measures including vitamin D status. *Photochemistry and Photobiology*.

[23] Nessvi S., Johansson L., Jopson J. (2011). Association of 25-hydroxyvitamin D3 levels in adult New Zealanders with ethnicity, skin color and self-reported skin sensitivity to sun exposure. *Photochemistry and Photobiology*.

[24] Al Shaikh A. M., Abaalkhail B., Soliman A. (2016). Prevalence of vitamin D deficiency and calcium homeostasis in Saudi children. *Journal of Clinical Research in Pediatric Endocrinology*.

[25] He S. Y., McCulloch C. E., Boscardin W. J., Chren M.-M., Linos E., Arron S. T. (2014). Self-reported pigmentary phenotypes and race are significant but incomplete predictors of Fitzpatrick skin phototype in an ethnically diverse population. *Journal of the American Academy of Dermatology*.

[26] Swiatoniowski A. K., Quillen E. E., Shriver M. D., Jablonski N. G. (2013). Technical note: comparing von Luschan skin color tiles and modern spectrophotometry for measuring human skin pigmentation. *American Journal of Physical Anthropology*.

[27] Tichondrias D. (2006). *File:Felix von Luschan Skin Color Chart.JPG Wilkipedia.org*.

[28] Florkowski C. M., Chew-Harris J. S. (2011). Methods of estimating GFR-different equations including CKD-EPI. *The Clinical Biochemist. Reviews*.

[29] Holick M. F., Binkley N. C., Bischoff-Ferrari H. A. (2011). Evaluation, treatment, and prevention of vitamin D deficiency: an Endocrine Society clinical practice guideline. *The Journal of Clinical Endocrinology & Metabolism*.

[30] NICE Guideline [NG34] Sunlight Exposure: Risks and Benefits, 2016, https://www.nice.org.uk/guidance/ng34

[31] Boucher B. J. (2012). The problems of vitamin d insufficiency in older people. *Aging and Disease*.

[32] Berridge M. J. (2017). Vitamin D deficiency and diabetes. *The Biochemical Journal*.

[33] Jeong H. Y., Park K. M., Lee M. J., Yang D. H., Kim S. H., Lee S.-Y. (2017). Vitamin D and hypertension. *Electrolyte Blood Press*.

[34] Van Buren P. N., Toto R. (2011). Hypertension in diabetic nephropathy: epidemiology, mechanisms, and management. *Advances in Chronic Kidney Disease*.

[35] Xiang F., Lucas R., de Gruijl F., Norval M. (2015). A systematic review of the influence of skin pigmentation on changes in the concentrations of vitamin D and 25-hydroxyvitamin D in plasma/serum following experimental UV irradiation. *Photochemical & Photobiological Sciences: Official Journal of the European Photochemistry Association and the European Society for Photobiology*.

[36] Ravnbak M. H. (2010). Objective determination of Fitzpatrick skin type. *Danish Medical Bulletin*.

[37] Shriver M. D., Parra E. J. (2000). Comparison of narrow-band reflectance spectroscopy and tristimulus colorimetry for measurements of skin and hair color in persons of different biological ancestry. *American Journal of Physical Anthropology*.

[38] Takiwaki H., Overgaard L., Serup J. (1994). Comparison of narrow-band reflectance spectrophotometric and tristimulus colorimetric measurements of skin color. *Skin Pharmacology and Physiology*.

[39] Treesirichod A., Chansakulporn S., Wattanapan P. (2014). Correlation between skin color evaluation by skin color scale chart and narrowband reflectance spectrophotometer. *Indian Journal of Dermatology*.

